# Treatment, outcomes, and demographics in sinonasal sarcoma: a systematic review of the literature

**DOI:** 10.1186/s12901-018-0052-5

**Published:** 2018-03-21

**Authors:** Mitchell R. Gore

**Affiliations:** Syracuse, USA

**Keywords:** Sinonasal sarcoma, Endoscopic, Survival, Kaplan-Meier, Sinonasal cancer

## Abstract

**Background:**

Sarcomas comprise a diverse group of soft tissue mesenchymal malignancies. The sinuses and nasal region are a relatively rare site of sarcomas.

**Methods:**

Retrospective review of the literature on sinonasal sarcomas from 1987-2017. Data were analyzed for demographics, treatment type, stage, and histopathologic type. Kaplan-Meier analysis was used to assess and compare survival.

**Results:**

A total of 198 cases of sinonasal sarcoma were identified and analyzed. The median age at diagnosis was 39 years. Overall 5-, 10-, and 20-year survival was 61.3%, 58.9%, and 49.1%, respectively, and disease-free 5-, 10-, and 20-year survival was 53.2%, 49.1%, and 38.3%, respectively. Lymph node metastasis was present at diagnosis in 3.0% of cases, and distant metastasis was present in 3.5% of cases. On univariate analysis T stage, overall stage, treatment type, histopathologic subtype, and presence of distant metastasis significantly affected survival. On multivariate analysis overall stage alone significantly predicted overall survival. Open vs. endoscopic surgery, total radiation dose, and presence of neck metastasis did not significantly affect survival. Combined modality treatment was associated with higher survival rates than single modality therapy.

**Conclusions:**

Sinonasal sarcoma is a relatively rare malignancy. Lower T and overall stage, lack of distant metastasis, and multimodality therapy were associated with improved survival. Certain histopathologic subtypes were associated with poorer survival.

**Electronic supplementary material:**

The online version of this article (10.1186/s12901-018-0052-5) contains supplementary material, which is available to authorized users.

## Background

Sarcomas of the head and neck region account for less than 10% of soft tissue sarcomas, and comprise less than 1% of head and neck malignancies [1, 2]. Sarcomas arise from mesenchymal tissue, and more than 50 histopathological subtypes of sarcoma have been reported. Approximately 80% of sarcomas arise from soft tissue, with the remaining originating from bone or cartilage. Head and neck sarcomas typically occur more frequently in men. While several recent studies have reported extensive patient data on specific histologic subtypes of sinonasal sarcoma [3–9], no recent study has examined the aggregate survival outcomes of the various subtypes of sarcoma found in the sinonasal cavity. Given the diverse histopathological subgroups comprising head and neck sarcomas, etiological factors have not been clearly elucidated in all subtypes. Some sarcoma subtypes are associated with known genetic factors, such as the translocation involving chromosomes X and 18 specific to synovial sarcoma, or the common t(11;22)(q24;q12) translocation present in the majority of Ewing’s sarcoma cases [7]. Sarcomas may be associated with diseases such as p53 mutations, basal cell nevus syndrome, Werner’s syndrome, tuberous sclerosis, neurofibromatosis, Gardner’s syndrome, and Li-Fraumeni syndrome. Previous irradiation for prior cancers or Epstein-Barr virus infection may also be associated with an increased risk of sarcoma. Most sarcomas are sporadic, not hereditary, in etiology [1–9].

Sarcomas of the sinonasal region may present a diagnostic challenge, as their location in the sinuses or nasal cavity may lead to presenting symptoms such as epistaxis, nasal congestion, or sinus pain and pressure that may be attributed to more benign causes such as chronic sinusitis, sinonasal polyposis, or allergic rhinitis. The less common sinonasal sarcoma subtypes may present a diagnostic challenge as their histopathological characteristics may overlap, especially with variations in tumor grade or with dedifferentiation. Additionally, the ideal treatment modality may present a therapeutic challenge, as the response to radiation and/or chemotherapy may vary according to the sarcoma subtype. Many patients are treated with multimodality therapy for sarcomas such as Ewing’s sarcoma, with surgery and/or radiation for local control, and chemotherapy for systemic treatment. Long-term surveillance of sinonasal sarcoma is necessary to monitor for and to detect recurrences and to allow salvage treatment if recurrence is detected.

Given the relative lack of literature on the aggregate survival outcomes and treatment in the diverse (and less common) histopathological types of sinonasal sarcoma, this study aimed to report the demographics, treatment modalities, and survival outcomes of patients treated for sinonasal sarcoma from studies published from 1987-2017. The study examined the correlation between survival outcomes and the AJCC (American Joint Committee on Cancer) sarcoma stage, tumor grade, presence of neck or distant metastasis, tumor type, treatment type, and endoscopic, endoscopic-assisted, or open surgery.

## Methods

### Systematic review and meta-analysis

**Fig. 1:**
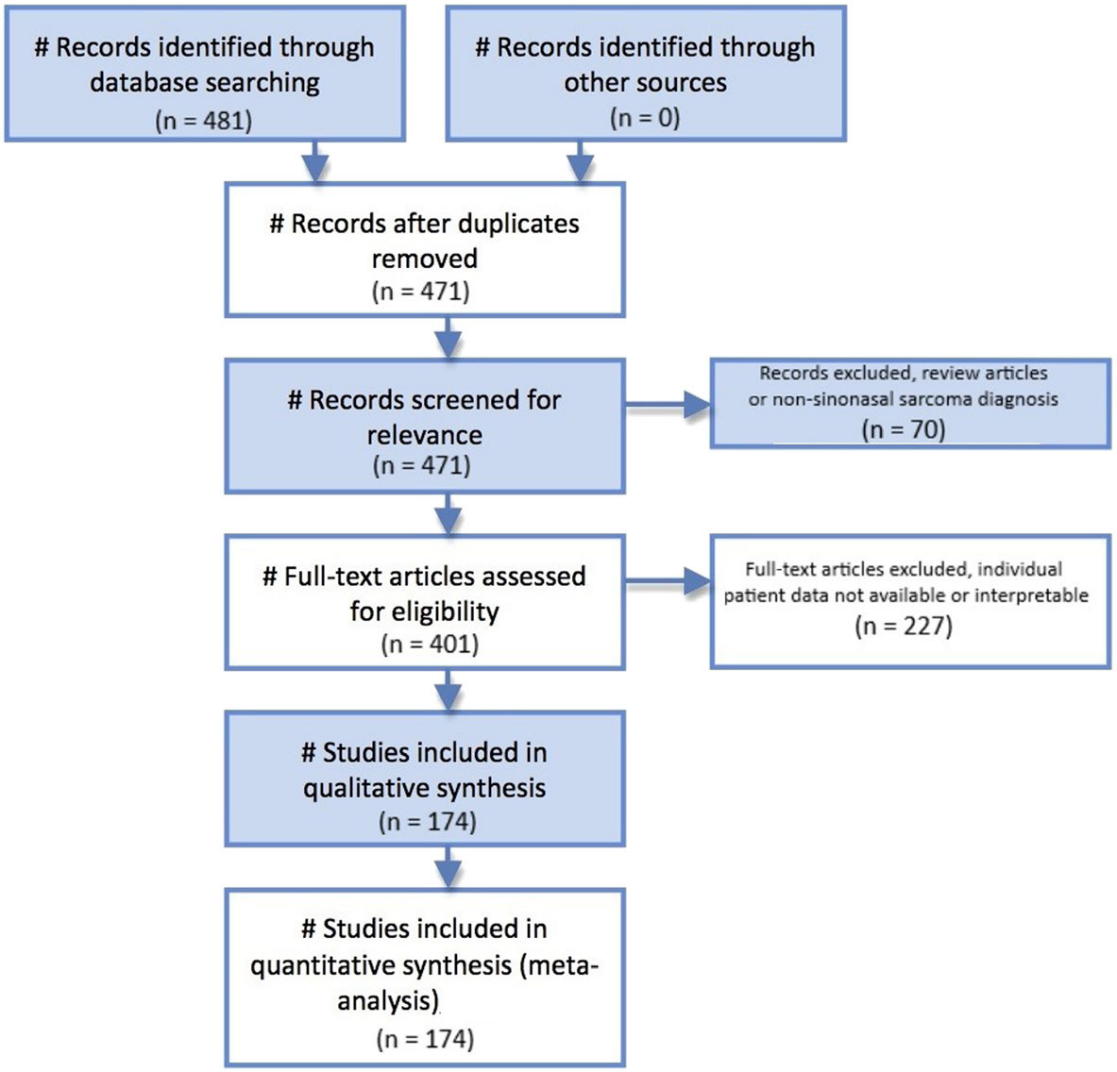
PRISMA diagram illustrating the methods for study selection

A Pubmed literature search was conducted using the search terms “sinus + nasal + sarcoma”. This literature review and meta-analysis was carried out and reported using the Preferred Reporting Items for Systematic Reviews and Meta-Analysis (PRISMA) Additional file 1 guidelines for the reporting of observational studies [10]. Figure 1 illustrates the PRISMA flow diagram for study selection. For the period 1987-2017 481 total papers were identified. After excluding duplicate studies, review articles, and studies without analyzable individual patient data, a total of 174 studies reporting 198 total patients were identified [11–184]. Case reports, case series, and cohort studies containing individual analyzable patient data on patients of any age with a pathologic diagnosis of sinonasal sarcoma were included in the meta-analysis of survival outcomes. The outcomes were overall survival and disease-free survival.

### Statistical analysis

Statistical analyses were performed with XLSTAT Biomed (Addinsoft, New York, NY, USA/Paris, France). Overall patient demographics, tumor histopathology, treatment factors, and outcomes were compiled via standard summary statistical methods. Tumors were staged according to the American Joint Committee on Cancer (AJCC) sarcoma staging system. Kaplan-Meier actuarial survival analysis and the log-rank test of statistical significance were performed to determine the univariate association between overall and disease-free survival and sarcoma T stage, overall sarcoma stage, treatment modality, neck metastasis, distant metastasis, tumor grade, total radiation dose < or >/= 50 Gray, and open, endoscopic assisted open, or endoscopic surgery. Linear regression was used to perform multivariate analysis. P values less than 0.05 were considered statistically significant.

## Results

### Demographics

One hundred ninety-eight patients were identified. Of patients for whom data on sex was available, 69.6% (128) were male, and 30.4% (56) were female (2.3:1 ratio). The average patient age was 38.4 years, the mean age was 39 years. Table 1 lists the sex, T, N, M, and stage demographics for the cohort. Table 2 lists the tumor type demographics. Table 3 lists the treatment types for the patients in the cohort. The histopathological diagnosis was alveolar soft part sarcoma in one patient (0.5%), angiosarcoma in four patients (2.0%), carcinosarcoma in two patients (1.0%), chondrosarcoma in sixteen patients (8.1%), Ewing’s sarcoma in fifty-six patients (28.3%), fibrosarcoma in five patients (2.5%), granulocytic sarcoma in five patients (2.5%), interdigitating dendritic cell sarcoma in one patient (0.5%), leiomyosarcoma in twelve patients (6.1%), liposarcoma in two patients (1.0%), low grade sinonasal sarcoma with neural and myogenic features in two patients (1.0%), myofibroblastic sarcoma in two patients (1.0%), myxofibrosarcoma in three patients (1.5%), neurofibrosarcoma in one patient (0.5%), neurosarcoma in one patient (0.5%), osteosarcoma in eleven patients (5.6%), peripheral neuroectodermal tumor (PNET) in fourteen patients (7.1%), rhabdomyosarcoma in fourteen patients (7.1%), sarcomatoid sarcoma in one patient (0.5%), synovial sarcoma in two patients (1.0%), teratocarcinosarcoma in fourteen patients (7.1%), teratosarcoma in one patient (0.5%), Triton tumor (malignant peripheral nerve sheath tumor with rhabdomyosarcomatous differentiation) in two patients (1.0%), and undifferentiated pleomorphic sarcoma/malignant fibrous histiocytoma (UPS/MFH) in 25 patients (12.6%).

**Table 1:** Sex, T, N, M, and overall stage demographics for the sinonasal sarcoma cohort

Characteristic	n	%
Male	128	69.6%
Female	56	30.4%
M+	7	3.5%
M0	191	96.5%
N+	6	3.0%
N0	192	97.0%
T1	113	57.1%
T2	85	42.9%
Stage I + Stage II	137	69.2%
Stage III	56	28.3%
Stage IV	5	2.5%

**Table 2:** Tumor type demographics for the sinonasal sarcoma cohort

Tumor Type	n	%
Alveolar soft part sarcoma	1	0.5%
Angiosarcoma	4	2.0%
Carcinosarcoma	2	1.0%
Chondrosarcoma	16	8.1%
Ewing’s sarcoma	56	28.3%
Fibrosarcoma	5	2.5%
Granulocytic sarcoma	5	2.5%
Interdigitating dendritic cell sarcoma	1	0.5%
Leiomyosarcoma	12	6.1%
Liposarcoma	2	1.0%
Low grade sinonasal sarcoma with neural and myogenic features	2	1.0%
Myofibroblastic sarcoma	2	1.0%
Myxofibrosarcoma	3	1.5%
Neurofibrosarcoma	1	0.5%
Neurosarcoma	1	0.5%
Osteosarcoma	11	5.6%
Peripheral neuroectodermal tumor (PNET)	14	7.1%
Rhabdomyosarcoma	14	7.1%
Sarcomatoid sarcoma	1	0.5%
Synovial sarcoma	2	1.0%
Teratocarcinosarcoma	14	7.1%
Teratosarcoma	1	0.5%
Triton tumor (malignant peripheral nerve sheath tumor with rhabdomyosarcomatous differentiation)	2	1.0%
Undifferentiated pleomorphic sarcoma/malignant fibrous histiocytoma (UPS/MFH)	25	12.6%

**Table 3:** Treatment type demographics for the sinonasal sarcoma cohort

Treatment	n	%
Surgery + radiation + chemotherapy	37	18.7%
Surgery + radiation	44	22.2%
Surgery + chemotherapy	17	8.6%
Surgery alone	45	22.7%
Radiation + chemotherapy	33	16.7%
Radiation alone	3	1.5%
Chemotherapy alone	10	5.1%
Palliative treatment	2	1.0%
No treatment	1	0.5%

### Survival analysis

**Fig. 2:**
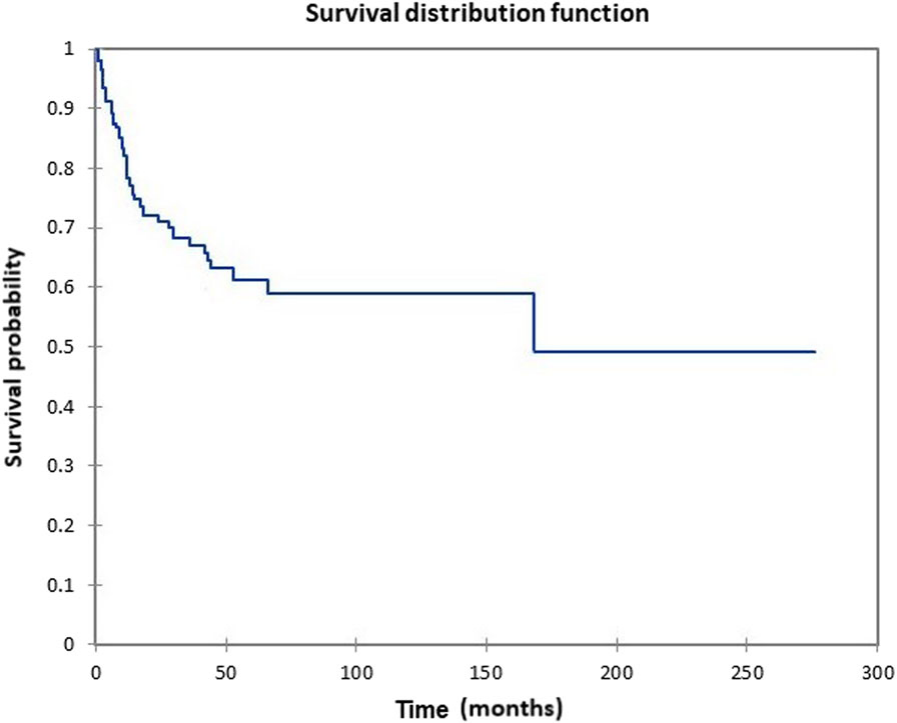
Kaplan-Meier overall survival for all patients

**Fig. 3:**
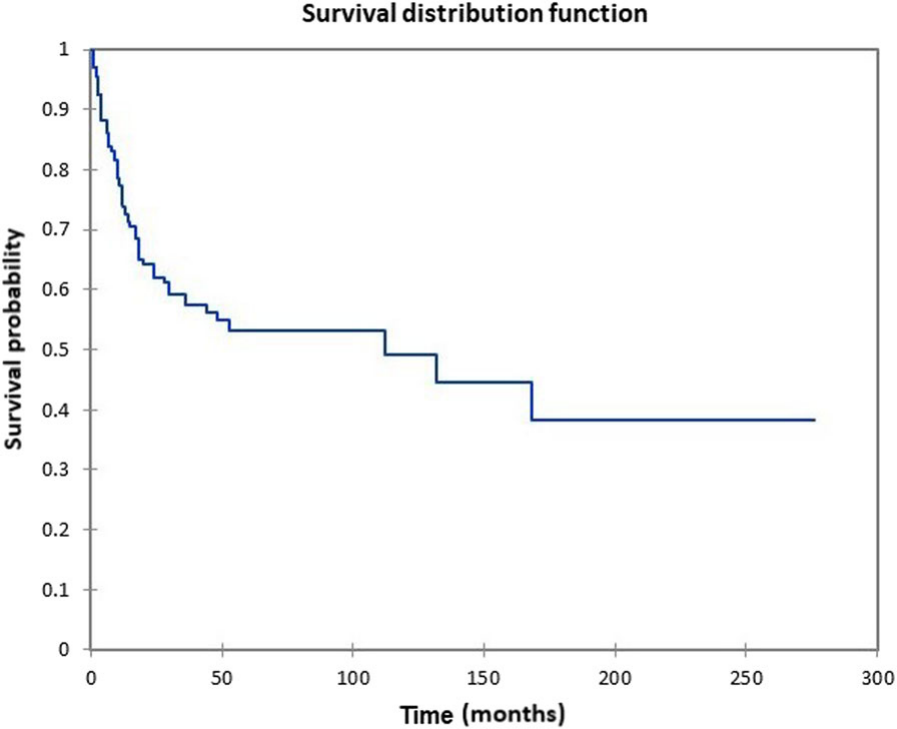
Kaplan Meier disease-free survival for all patients

The site of tumor involvement was the nasal cavity in 110 patients, maxillary sinus in 115 patients, ethmoid sinus in 55 patients, sphenoid sinus in 20 patients, frontal sinus in 20 patients, the orbit in 54 patients, the dura in 7 patients, brain in 4 patients, the cribriform plate in 7 patients, the cavernous sinus in 3 patients, and the sella turcica in 5 patients (numbers > 198 due to individual patients having more than one site of tumor involvement). Seven patients (3.5%) had distant metastasis (M+) at the time of diagnosis, and six patients (3.0%) had cervical metastasis (N+) at the time of diagnosis. Overall survival (OS) at 60 months, 120 months, and 240 months was 61.3%, 58.9%, and 49.1%, respectively (Fig. 2), with a mean overall actuarial survival of 130.6 months. Disease-free survival (DFS) at 60 months, 120 months, and 240 months was 53.2%, 49.1%, and 38.3%, respectively (Fig. 3), with a mean disease-free actuarial survival of 111.2 months. Survival was improved for patients who did not experience disease recurrence, and the relative survival in patients who did not experience a recurrence (*n* = 122) vs. patients who experienced a recurrence (*n* = 76) was 2.5:1 (*p* = 0.0001).

**Fig. 4:**
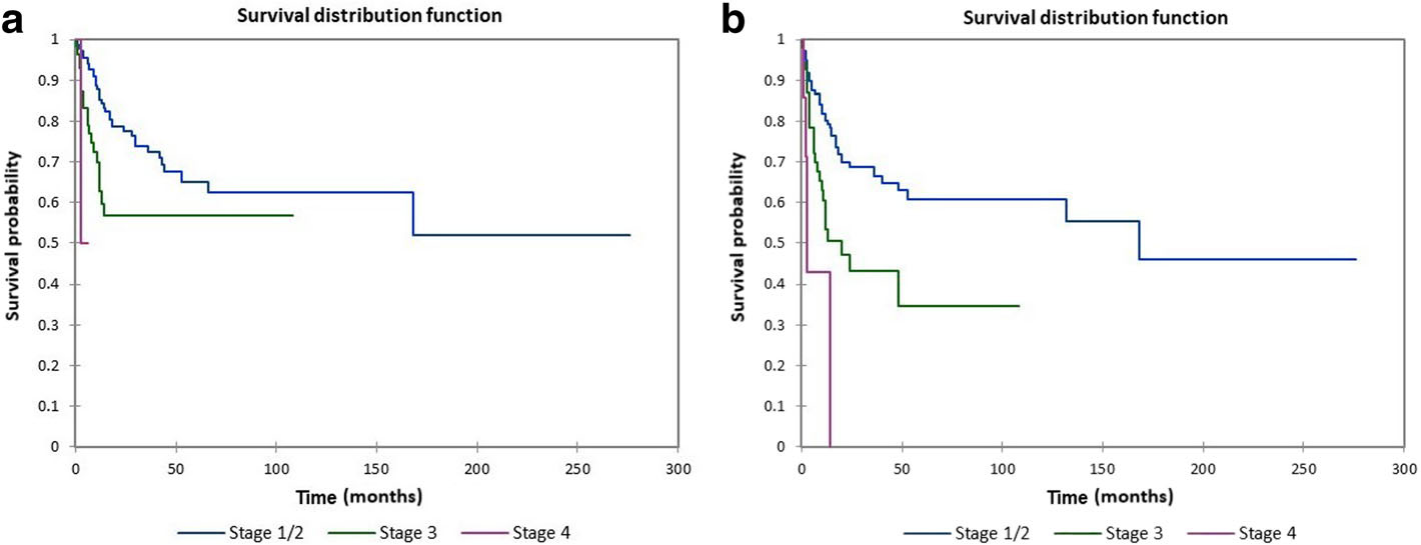
**a** Kaplan-Meier overall survival by stage. **b** Kaplan-Meier disease-free survival by stage

Figure 4a shows the Kaplan-Meier actuarial overall survival by stage, while Fig. 4b shows the Kaplan-Meier actuarial disease-free survival by stage. Overall survival at 60 months was 65.5% for stage I + stage II (*n* = 137), 56.8% for stage III (*n* = 56), and 0% for stage IV (*n* = 5), *p* = 0.001. Disease-free survival at 60 months was 60.8% for stage I + stage II (*n* = 137), 34.6% for stage III (*n* = 56), and 0% for stage IV (*n* = 5), *p* < 0.0001.

**Fig. 5:**
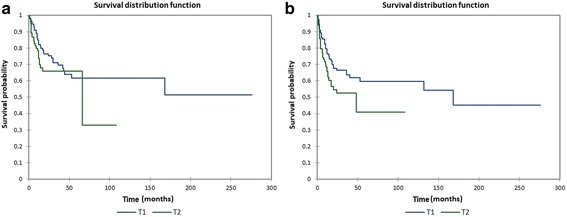
**a** Kaplan-Meier overall survival by tumor (T) stage. **b** Kaplan-Meier disease-free survival by tumor (T) stage

Figure 5a shows the Kaplan-Meier actuarial overall survival by tumor (T) stage, while Fig. 5b shows the Kaplan-Meier actuarial disease-free survival by tumor (T). Overall survival at 60 months was 61.7% for T1 (*n* = 113), and 65.8% for T2 (*n* = 85), *p* = 0.2. Disease-free survival at 60 months was 59.6% for T1 (*n* = 113), and 40.8% for T2 (*n* = 85), *p* = 0.04.

**Fig. 6:**
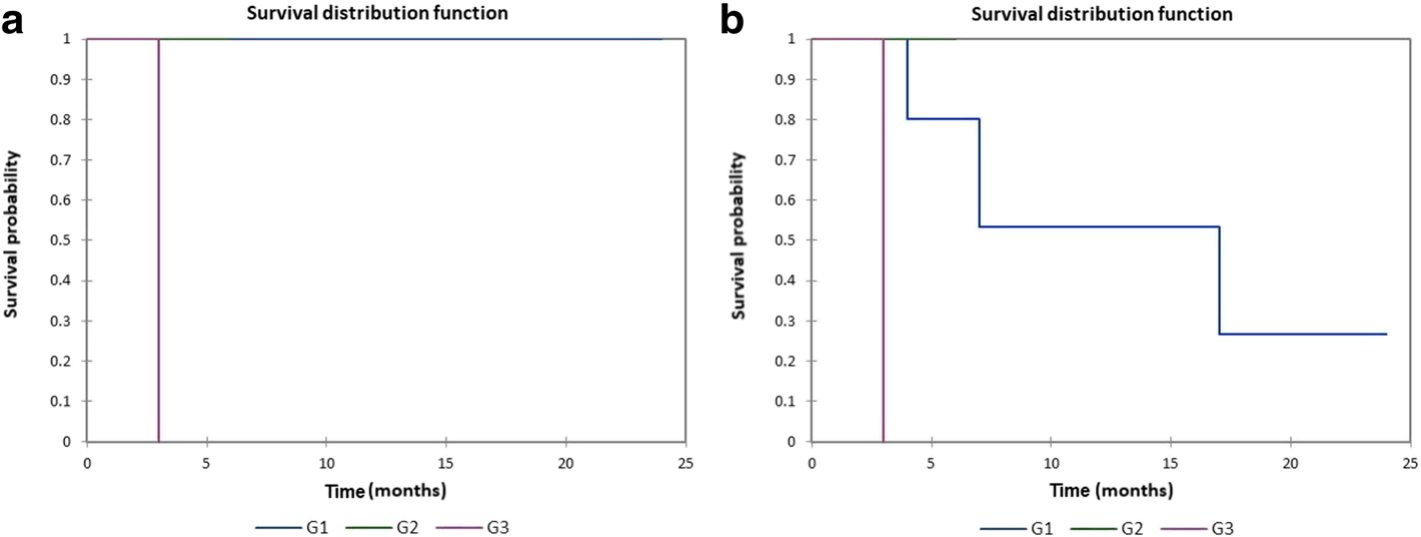
**a** Kaplan-Meier overall survival by tumor grade (G). **b** Kaplan-Meier disease-free survival by tumor grade (G)

**Fig. 7:**
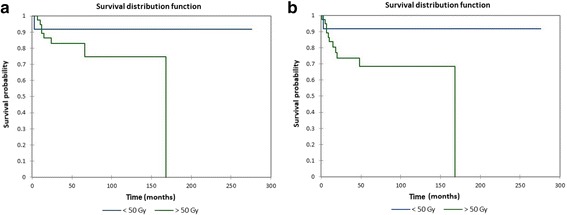
**a** Kaplan-Meier overall survival by total radiation dose. **b** Kaplan-Meier disease-free survival by total radiation dose

Figure 6a shows the Kaplan-Meier actuarial overall survival by tumor grade (G), while Fig. 6b shows the Kaplan-Meier actuarial disease-free survival by tumor grade. Mean overall survival for grade 1 (*n* = 5) was 12 months, grade 2 was 6 months (*n* = 1), and grade 3 was 3 months (*n* = 1), *p* = 0.05, while mean disease-free survival for grade 1 was 11.0 months (*n* = 5), grade 2 was 6 months (*n* = 1), and grade 3 was 3 months (*n* = 1), *p* = 0.05. Figure 7a shows the Kaplan-Meier actuarial overall survival by radiation dose, while Fig. 7b shows the Kaplan-Meier actuarial disease-free survival by radiation dose. Overall survival at 60 months was 91.7% for <50 Gray (*n* = 12), and 82.9% for >/= 50 Gray (*n* = 42, *p* = 0.2). Disease-free survival at 60 months was 91.7% for <50 Gray (*n* = 12), and 68.9% for >/= 50 Gray (*n* = 42, *p* = 0.1).

**Fig. 8:**
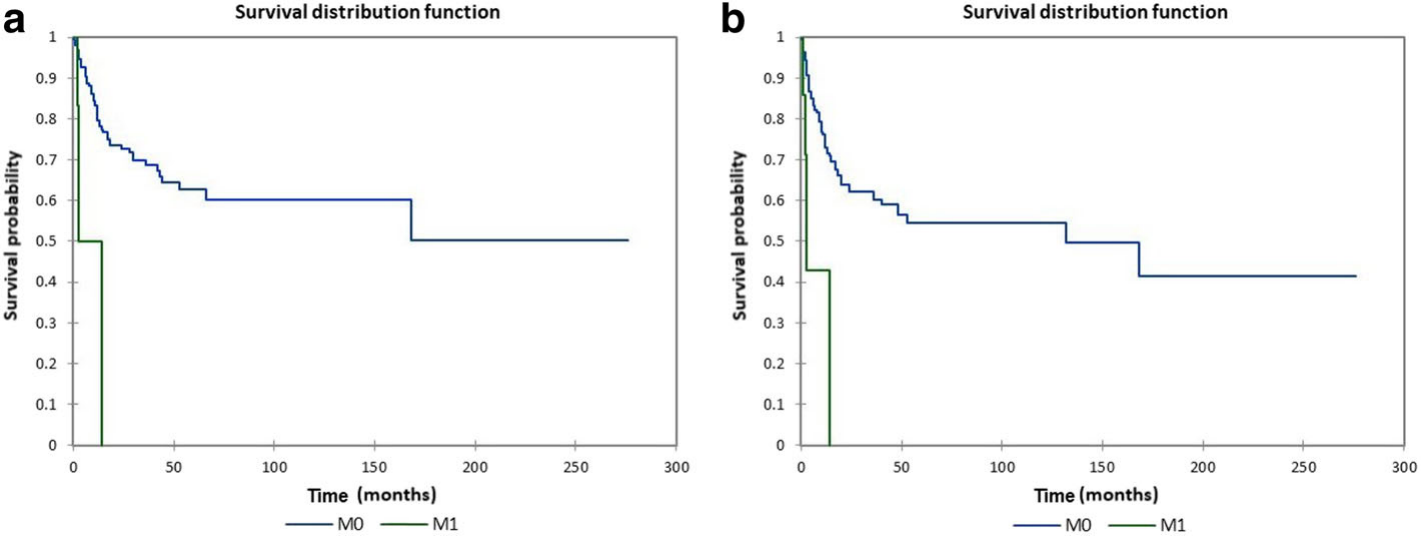
**a** Kaplan-Meier overall survival by metastasis (M) stage. **b** Kaplan-Meier disease-free survival by metastasis (M) stage

**Fig. 9:**
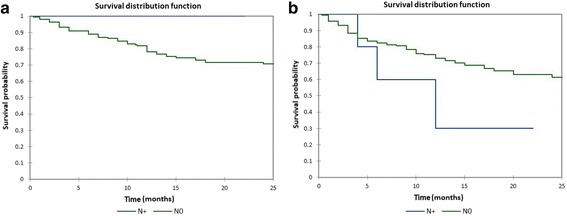
**a**. Kaplan-Meier overall survival by neck (N) stage. **b** Kaplan-Meier disease-free survival by neck (N) stage

Figure 8a shows the Kaplan-Meier actuarial overall survival for M0 (no distant metastasis) vs. M1 (distant metastasis present) patients, while Fig. 8b shows the Kaplan-Meier actuarial disease-free survival for M0 vs. M1 patients. Overall survival at 60 months was 62.7% for M0 patients (*n* = 191), and 0% for M1 patients (*n* = 7), *p* < 0.0001. Disease-free survival at 60 months was 54.6% for M0 patients (*n* = 191), and 0% for M1 patients (*n* = 7), *p* < 0.0001. Figure 9a shows the Kaplan-Meier actuarial overall survival for N0 (no neck metastasis) vs. N+ (neck metastasis present) patients, while Fig. 9b shows the Kaplan-Meier actuarial disease-free survival for N0 vs. N+ patients. Overall survival at 60 months was 61.0% for N0 patients (*n* = 192), and NA (not available) for N+ patients (*n* = 6), *p* = 0.3. Disease-free survival at 60 months was 53.8% for N0 patients (*n* = 192), and NA for N+ patients (*n* = 6), *p* = 0.2.

**Fig. 10:**
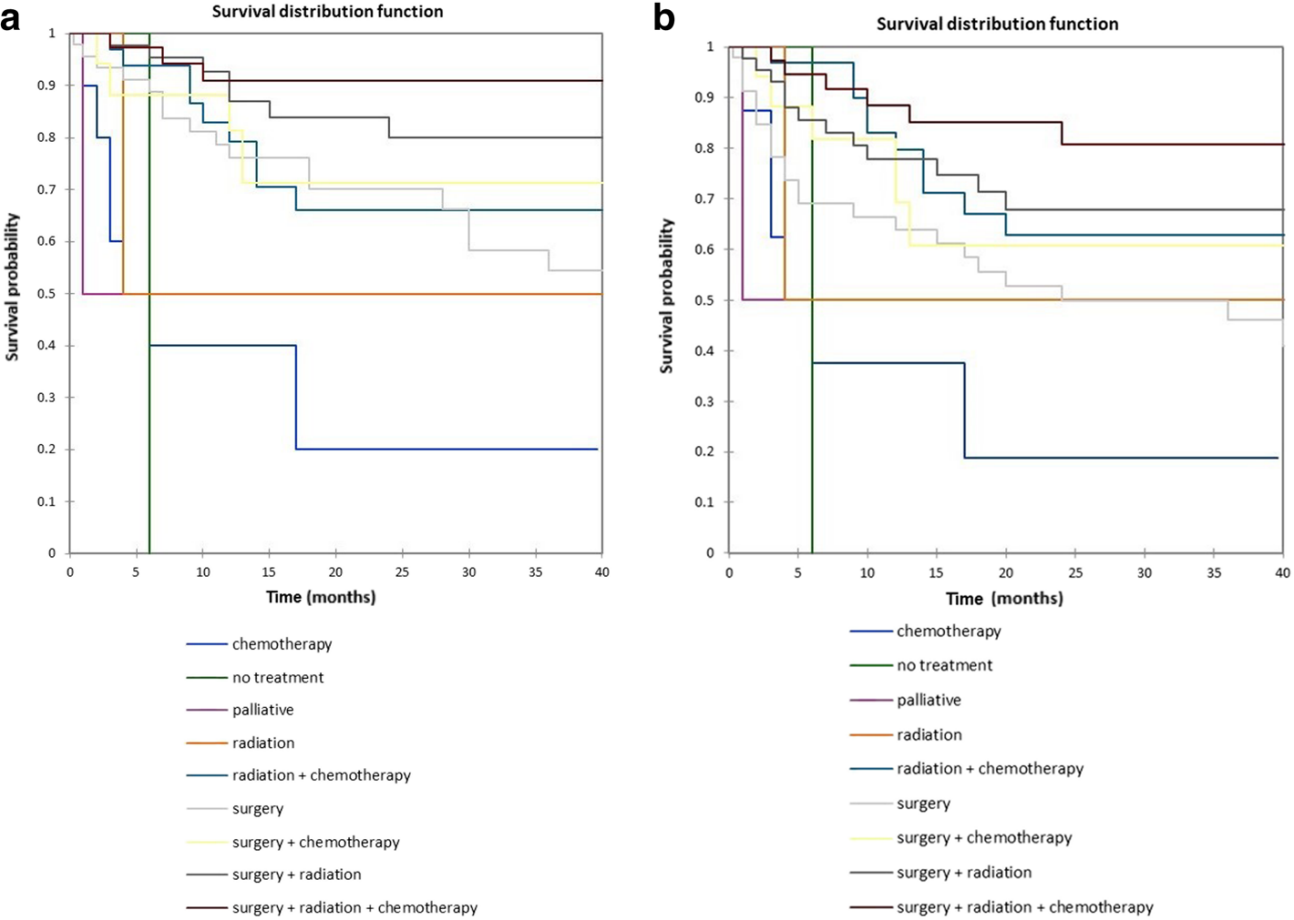
**a** Kaplan-Meier overall survival by treatment modality. **b** Kaplan-Meier disease-free survival by treatment modality

Figure 10a shows the Kaplan-Meier actuarial overall survival by treatment modality, while Fig. 10b shows the Kaplan-Meier actuarial disease-free survival by treatment modality. Overall survival at 60 months was 90.9% for surgery + radiation + chemotherapy (*n* = 37), 79.9% for surgery + radiation (*n* = 44), 71.3% for surgery + chemotherapy (*n* = 17), 40.9% for surgery alone (*n* = 45), 66% for radiation + chemotherapy (*n* = 33), NA for radiation alone (*n* = 3) and chemotherapy alone (*n* = 10), and 0% for palliative treatment (*n* = 2) and no treatment (*n* = 1), *p* < 0.0001. Disease-free survival at 60 months was 80.8% for surgery + radiation + chemotherapy (*n* = 37), 67.9% for surgery + radiation (*n* = 44), 60.7% for surgery + chemotherapy (*n* = 17), 40.9% for surgery alone (*n* = 45), 57.2% for radiation + chemotherapy (*n* = 33), NA for radiation alone (*n* = 3) and chemotherapy alone (*n* = 10), and 0% for palliative treatment (*n* = 2) and no treatment (*n* = 1), *p* < 0.0001.

**Fig. 11:**
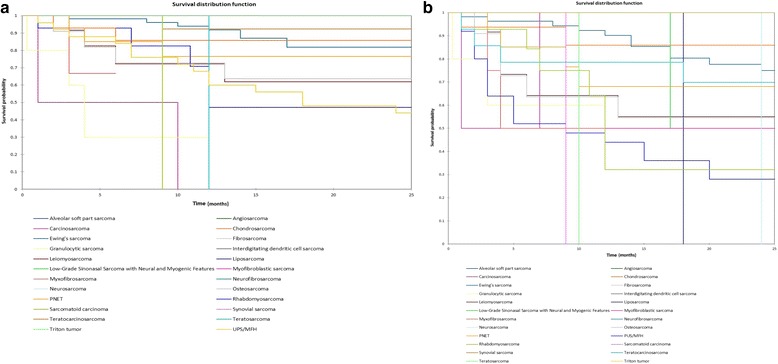
**a** Kaplan-Meier overall survival by tumor type. **b** Kaplan-Meier disease-free survival by tumor type

Figure 11a shows the Kaplan-Meier actuarial overall survival by histopathological tumor type, while Fig. 11b shows the Kaplan-Meier actuarial disease-free survival by histopathological tumor type. Overall survival at 60 months was greatest for chondrosarcoma (92.3%, *n* = 16), Ewing’s sarcoma (81.8%, *n* = 56), osteosarcoma (63.6%, *n* = 11), PNET (76.6%, *n* = 14), teratocarcinosarcoma (68.6%, *n* = 14), myofibroblastic sarcoma (100%, *n* = 2), Triton tumor (100%, *n* = 2), leiomyosarcoma (61.9%, *n* = 12), and worse for carcinosarcoma (0%, *n* = 2), neurosarcoma (0%, *n* = 1), rhabdomyosarcoma (47.2%, *n* = 14), sarcomatoid sarcoma (0%, *n* = 1), teratosarcoma (0%, *n* = 1), PUS/MFH (24%, *n* = 25), and NA for angiosarcoma (*n* = 4), fibrosarcoma (*n* = 5), granulocytic sarcoma (*n* = 5), interdigitating dendritic cell sarcoma (*n* = 1), liposarcoma (*n* = 2), myxofibrosarcoma (*n* = 3), neurofibrosarcoma (*n* = 1), and synovial sarcoma (*n* = 2), and alveolar soft part sarcoma (*n* = 1), *p* < 0.0001. Disease-free survival at 60 months was greatest for chondrosarcoma (86%, *n* = 16), Ewing’s sarcoma (75%, *n* = 56), osteosarcoma (54.5%, *n* = 11), PNET (68.1%, *n* = 14), teratocarcinosarcoma (47%, *n* = 14), myofibroblastic sarcoma (50%, *n* = 2), Triton tumor (50%, *n* = 2), and worse for carcinosarcoma (0%, *n* = 2), neurosarcoma (0%, *n* = 1), sarcomatoid sarcoma (0%, *n* = 1), teratosarcoma (0%, *n* = 1), PUS/MFH (20%, *n* = 25), and NA for angiosarcoma (*n* = 4), fibrosarcoma (*n* = 5), granulocytic sarcoma (*n* = 5), interdigitating dendritic cell sarcoma (*n* = 1), liposarcoma (*n* = 2), myxofibrosarcoma (*n* = 3), neurofibrosarcoma (*n* = 1), and synovial sarcoma (*n* = 2), leiomyosarcoma (*n* = 12), rhabdomyosarcoma (*n* = 14), and alveolar soft part sarcoma (*n* = 1), *p* < 0.0001.

**Fig. 12:**
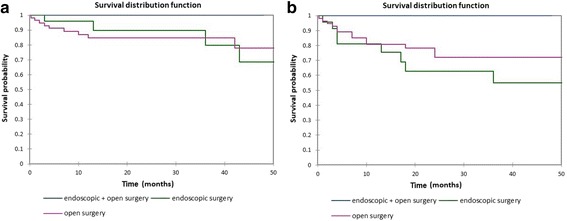
**a** Kaplan-Meier overall survival by type of surgery. **b** Kaplan-Meier disease-free survival by type of surgery

Figure 12a shows the Kaplan-Meier actuarial overall survival for endoscopic assisted open surgery (endoscopic + open) vs. open surgery vs. endoscopic surgery, while Fig. 12b shows the Kaplan-Meier actuarial disease-free survival for endoscopic assisted open surgery (endoscopic + open) vs. open surgery vs. endoscopic surgery. Overall survival at 60 months was 100% for endoscopic assisted open surgery (*n* = 3), 77.8% for open surgery (*n* = 57), and 68.5% for endoscopic surgery (*n* = 24), *p* = 0.8. Disease-free survival at 60 months was 100.0% for endoscopic assisted open surgery (*n* = 3), 72.0% for open surgery (*n* = 57), and 55% for endoscopic surgery (*n* = 24), *p* = 0.4.

Linear regression was utilized to conduct a multivariate analysis for overall and disease-free survival. On multivariate analysis overall stage alone was a significant predictor of overall survival (*p* < 0.0001). On multivariate analysis overall stage was not a significant predictor of disease-free survival (*p* = 0.1).

## Discussion

Sarcomas of the head and neck are relatively uncommon, accounting for approximately 10% or less of head and neck tumors. The sinonasal region is even more uncommon, with rhabdomyosarcoma of the sinonasal region occurring in approximately 0.034/100,000 persons. In the present study the mean age was 39, with a male to female ratio of 2.3:1. In their study of head and neck sarcomas in patients treated at St. Thomas’ NHS (National Health Service) Foundation Trust from 1996-2012 Stavrakas et al. [2] noted a mean age of 62 and a male to female ratio of approximately 2:1.

The present study showed a significant relationship on univariate analysis between actuarial survival and tumor histopathological type (OS and DFS), presence of distant metastasis (OS and DFS), treatment type (OS and DFS), and T stage (DFS). Tumor grade approached significance despite low numbers of patients with specific tumor grade reported (OS and DFS). Open vs. endoscopic assisted vs. endoscopic surgery, radiation dose < or >/= 50 Gray, and presence of neck metastasis was not significantly associated with overall or disease-free survival on univariate analysis. The overall and disease-free survival at 60 months for all patients was 61.3% and 53.2%, respectively. This is higher than the overall (25.6%) and disease-free (25.6%) 60-month survival rates noted for the head and neck sarcoma cohort reported by Stavrakas et al. [2], of which 23.0% were sinonasal sarcomas. The sinonasal group in the Stavrakas study [2] had a 60-month actuarial disease-specific survival of approximately 55%, which is similar to the disease-free survival noted in the present study.

Stephan et al. [3] and Unsal et al. [4] reported outcomes for adult patients with sinonasal rhabdomyosarcoma identified from the National Cancer Database and patients with sinonasal rhabdomyosarcoma identified in the Surveillance, Epidemiology, and End Results (SEER) database, respectively. Stephan et al. [3] noted positive regional nodes in 84.6% of patients and distant metastasis in 27.7% of patients, while Unsal et al. [4] noted positive regional nodes in 54.3% of patients and distant metastasis in 32.2%. The present study noted much lower rates of distant (3.5%) and neck (3.1%) metastasis. Stephan et al. [3] and Unsal et. al. [4] also noted lower 60-month overall survival rates (28.4% and 35.1%, respectively) than the present study (61.3%). In the present study the 60-month survival for the 14 patients with sinonasal rhabdomyosarcoma was 47.2%, which was lower than the actuarial 60-month survival for the entire cohort. The lower survival in the Stephan [3] and Unsal [4] studies is thus likely a combination of a poorer prognosis for sinonasal rhabdomyosarcoma compared to some of the more favorable sarcomas included in the present study such as Ewing’s sarcoma and chondrosarcoma, and the higher neck and distant metastasis rate in sinonasal rhabdomyosarcoma vs. the overall cohort in the present study including several less aggressive sarcomas. Stephan et al. [3] noted a significantly lower 60-month survival for patients with distant metastasis (~18%) vs. M0 patients (~35%), similar to the significantly decreased 60-month overall and disease-free survival seen in M1 patients in the present study (both 0% at 60 months). Interestingly, Unsal et al. [4] noted significantly lower 60-month survival for patients with distant metastasis but not for patients with regional lymph node metastasis, similar to the findings in the present study.

Combined modality therapy was associated with increased 60-month survival vs. single modality therapy. Sixty-month overall survival rates for surgery + radiation + chemotherapy (90.9%), surgery + radiation (79.9%), surgery + chemotherapy (71.3%), and radiation + chemotherapy (66.0%) were significantly higher than surgery (40.9%), radiation (NA), and chemotherapy (NA) (*p* < 0.0001). Sixty-month disease free survival rates for surgery + radiation + chemotherapy (80.2%), surgery + radiation (68.8%), surgery + chemotherapy (57.7%), and radiation + chemotherapy (58.4%) were significantly higher than surgery (39.6%), radiation (NA), and chemotherapy (NA)(*p* < 0.0001). Stavrakas [2] also noted higher 60-month survival rates for patients treated with adjuvant therapy vs. surgery alone. In their study of 5 patients with sinonasal Ewing’s sarcoma Lombardi et al. [185] noted only one death, due to distant metastases, with all of the 5 patients being treated with multimodality treatment.

Ewing’s sarcoma was the most frequent histopathologic type, with 56 patients (28%), followed by UPS/MFH with 25 (12.6%), chondrosarcoma with 16 (8.0%), and rhabdomyosarcoma (14, 7.0%), PNET (14, 7.0%), teratocarcinosarcoma (14, 7.0%), leiomyosarcoma, (12, 6.0%), and osteosarcoma (11, 5.6%). Stavrakas et al. [2] noted sinonasal sarcoma to be the most frequent subsite in their study (23.0%), with Kaposi sarcoma in 20.5%, chondrosarcoma in 15.3% of patients and osteosarcoma in 10.2%, and leiomyosarcoma, dermatofibrosarcoma, and spindle cell sarcoma in 7.6%. On univariate analysis the present study showed a significant association between improved overall and disease-free survival for Ewing’s sarcoma, chondrosarcoma, PNET, teratosarcoma, osteosarcoma, leiomyosarcoma, Triton tumor, and myofibroblastic sarcoma vs. UPS/MFH, sarcomatoid sarcoma, rhabdomyosarcoma, neurosarcoma, and carcinosarcoma. The prevalence of Ewing’s sarcoma, chondrosarcoma, PNET, teratocarcinosarcoma, osteosarcoma, and leiomyosarcoma in the present study likely contributes to the higher overall and disease-free survival noted in this study vs. the sinonasal rhabdomyosarcoma groups seen in the Stepan [3] and Unsal [4] studies.

Only approximately 3.5% of patients in the present study had tumor grade information reported. This is lower than the approximately 20.0% of cases with grade information noted in the Unsal et. al. study [4], but is consistent with a minority of reported cases having specific tumor grade information reported. Unsal et al. [4] proposed that this low number may be due to the heterogeneous pathologic features seen in sarcomas, and the use of histologic subtype as a de facto surrogate for grade (i.e. embryonal vs. alveolar). Despite the low numbers of cases with grade reported in the present study, grade 1 tumors displayed a mean survival (~11.0 months) that was longer than grade 2 (mean 6.0 months) or grade 3 (mean 3.0 months) tumors. This difference approached significance (*p* = 0.05 for OS and *p* = 0.05 for DFS). Radiation dose greater or less than 50 Gray and endoscopic vs. endoscopic assisted open vs. open surgery did not have a significant association with survival.

Unsal et al. [4] noted lower 60-month survival for Intergroup Rhabdomyosarcoma Staging Group (IRSG) stage IV patients vs. stage III or II patients, similar to the present study. In the present study overall stage using the AJCC sarcoma staging system was significantly associated with survival, with stage IV patients having significantly lower overall survival than stage III or stage I/II patients. This significant overall survival difference remained significant on multivariate analysis.

The present study has several limitations. The diverse group of histopathological subtypes included in the analysis has the potential to affect the results, adding heterogeneity that would be less prevalent in an analysis of a specific sarcoma subtype. Additionally, the retrospective data introduces the potential for selection and recall bias. Given the relative rarity of sinonasal sarcoma it would be more difficult to assemble randomized controlled trials specific to sinonasal sarcoma that would reduce or eliminate the inherent selection bias. Additionally, the limited available data on tumor grade limits the analysis of tumor grade as a prognostic factor, although even with the low numbers in the present study tumor grade approached significance on univariate analysis.

Overall, the study suggests that combined modality therapy is associated with improved outcomes, and suggests that type of surgery (endoscopic vs. open) does not appear to affect survival. Additionally, the rate of positive cervical nodes at diagnosis was low (3.0%) in the present study, suggesting that the clinically and radiographically negative neck may not warrant empirical treatment in sinonasal sarcoma. Tumor type and overall stage were significantly associated with survival, making accurate histopathological diagnosis and accurate staging vital. The strengths of this study include the relatively large cohort and the univariate and multivariate survival analysis, and the long follow-up data available on the present cohort, as well as the detailed data collected on tumor types, TNM and overall staging, radiation dose and surgery type. Future prospective studies and/or population-based studies would further add to the data on the effect of treatment type, stage, grade, and tumor histopathology on survival. Additionally, single-tumor-type studies on the more common histopathological subtypes in sinonasal sarcoma may further elucidate treatment and survival characteristics unique to individual subtypes.

## Conclusions

Head and neck sarcoma is a rare entity, comprising ~ 10% or less of all sarcomas, with sinonasal sarcomas likely representing only 25% or less of head and neck sarcomas. The present study demonstrated a significant association between overall stage and overall survival on univariate and multivariate analysis, and overall stage appeared to be the most significant contributor to survival in the present study. On univariate analysis overall stage, T stage, combined modality vs. single modality therapy, distant metastasis, and tumor subtype all showed a significant correlation with survival. Surgical excision with negative margins when possible combined with adjuvant radiation and/or chemotherapy appears to offer the best survival outcomes. Especially for more favorable tumor subtypes such as Ewing’s, PNET, chondrosarcoma, teratocarcinosarcoma, and Triton tumor long-term survival is possible, especially with combined modality therapy.

## Additional file

Additional file 1: PRISMA 2009 checklist for Treatment, Demographics, and Outcomes in Sinonasal Sarcoma: a Systematic Review of the Literature. (DOC 63 kb)

## Abbreviations

AJCC: American Joint Committee on Cancer
DFS: Disease-free survival
G: Grade
M: Metastasis
N: Neck
NA: Not available
OS: Overall survival
PNET: Primitive Neuroectodermal Tumor
PRISMA: Preferred Reporting Items for Systematic Reviews and Meta*-*Analyses
SEER: Surveillance, Epidemiology, and End Results
T: Tumor
UPS/MFH: Undifferentiated Pleomorphic Sarcoma/Malignant Fibrous Histiocytoma
